# Influence of Sequential vs. Simultaneous Dual-Task Exercise Training on Cognitive Function in Older Adults

**DOI:** 10.3389/fnagi.2017.00368

**Published:** 2017-11-07

**Authors:** Jamie L. Tait, Rachel L. Duckham, Catherine M. Milte, Luana C. Main, Robin M. Daly

**Affiliations:** ^1^Institute for Physical Activity and Nutrition, School of Exercise and Nutrition Sciences, Deakin University, Geelong, VIC, Australia; ^2^Australian Institute for Musculoskeletal Science, St. Albans, Melbourne, VIC, Australia

**Keywords:** dual-task training, cognition, aging, physical activity, older adults

## Abstract

Emerging research indicates that exercise combined with cognitive training may improve cognitive function in older adults. Typically these programs have incorporated sequential training, where exercise and cognitive training are undertaken separately. However, simultaneous or dual-task training, where cognitive and/or motor training are performed simultaneously with exercise, may offer greater benefits. This review summary provides an overview of the effects of combined simultaneous vs. sequential training on cognitive function in older adults. Based on the available evidence, there are inconsistent findings with regard to the cognitive benefits of sequential training in comparison to cognitive or exercise training alone. In contrast, simultaneous training interventions, particularly multimodal exercise programs in combination with secondary tasks regulated by sensory cues, have significantly improved cognition in both healthy older and clinical populations. However, further research is needed to determine the optimal characteristics of a successful simultaneous training program for optimizing cognitive function in older people.

## Introduction

Age-associated cognitive decline, which can progress to mild cognitive impairment and dementia, are growing public health concerns with no known cure. Regular exercise has been shown to provide some cognitive benefits in healthy and cognitively impaired older adults (Colcombe and Kramer, 2003; Heyn et al., 2004; Angevaren et al., 2008), but the optimal type and dose (frequency, intensity, duration) of exercise remains unclear. Cognitive training and regular participation in mentally stimulating activities have also been demonstrated to have positive effects on cognitive function in older adults (Valenzuela and Sachdev, 2009; Martin et al., 2011), but there is uncertainty as to whether such training alone can enhance functional tasks of everyday living such as talking whilst walking. A reduced ability to simultaneously divide attention between a primary and secondary task, which is referred to as the dual-task paradigm, is clinically important as it has been associated with reduced reaction time and walking speed, more frequent contact with obstacles whilst walking, and an increased risk of falls (Melzer et al., 2010; Pichierri et al., 2011). In conjunction with age-associated deterioration in executive function, deficits in divided attention can be problematic for tasks such as driving (Anstey et al., 2005), and represents a strong risk factor for falls in the elderly (Tinetti et al., 1995; Muir et al., 2012), which collectively increase the risk for institutionalization (Tinetti and Williams, 1997; Bharucha et al., 2004). Given this research, there has been recent interest into whether dual-task cognitive-motor exercise training, whereby cognitive or motor tasks are performed sequentially or simultaneously with exercise training, result in greater improvements in selected cognitive domains than either exercise or cognitive training alone to prolong functional independence, or reduce the risk of cognitive-related diseases such as dementia and Alzheimer's disease. This review will provide an overview of the evidence related to the effectiveness of sequential (separate) vs. simultaneous exercise-cognitive training for improving cognitive function, and whether there is an optimal modality and dose for cognitive gains in healthy and cognitively impaired older adults. Specifically, this review will include evidence from 29 randomized controlled trials (RCTs) and factorial design studies ≥7 weeks in duration and published up to December 2016, and will extend on previous reviews that have not delineated the specific benefits from simultaneous and sequential studies (Law et al., 2014; Lauenroth et al., 2016).

## Effects of sequential exercise-cognitive training on cognitive function

An overview of the trials which have investigated whether combined sequential cognitive-exercise training can benefit cognitive function in older adults to a greater extent than either cognitive or exercise training alone is provided in Table 1. While there is some evidence that exercise training followed sequentially by cognitive/motor training on the same day (prior to or after exercise training), or separate days, can enhance cognitive abilities including memory and executive function in healthy older adults (Schaefer and Schumacher, 2011; Law et al., 2014; Lauenroth et al., 2016), there is considerable heterogeneity between the different interventions which makes interpretation of the results difficult. In one of the first factorial designed randomized controlled trials (RCT) to address whether sequential exercise-cognitive training improved cognitive function in healthy older adults, Fabre et al. (2002) found that 8-weeks of moderate-intensity aerobic training performed twice per week combined with memory training once per week was more effective at improving a composite memory score than either approach alone. However, a limitation of this study was that the total dose of training was greater in the combined training group, compared to each intervention alone. Despite this, a 16-week factorial design RCT in 180 adults aged 65–93 years living independently in retirement villages found that a combination of walking and progressive resistance training (PRT), and cognitive training (each performed three times per week), or cognitive training alone, led to greater improvements in hand-eye coordination, global visual memory, and processing speed, than those who did not engage in cognitive training (exercise-only and controls; Shatil, 2013). However, as cognitive outcomes assessed in this trial were also included in cognitive training (both used the CogniFit program), these measures may lack external or clinical validity (Noack et al., 2014; Ratner and Atkinson, 2015; Simons et al., 2016). As shown in Table 1 the findings from a number of other trials have demonstrated that sequential training involving interventions ranging from 7 to 30 weeks, and incorporating a range of intensities of aerobic and/or PRT in combination with cognitive training, did not confer any superior transfer effects to cognitive performance compared to cognitive training alone (Oswald et al., 2006; Linde and Alfermann, 2014; Shah et al., 2014; Rahe et al., 2015; Desjardins-Crépeau et al., 2016). Furthermore, few studies have demonstrated greater cognitive benefits following sequential training compared to exercise alone in older adults aged >50 years (Fabre et al., 2002; Shatil, 2013; van het Reve and de Bruin, 2014).

**Table 1:** Randomized controlled trials examining the effects of sequential exercise-cognitive training on cognitive function in older adults.

**Author**	**Participants**	**N**	**Study design**	**Duration (weeks)**	**Cognitive intervention**		**Exercise intervention**		**Control group**	**Cognitive outcomes ExCT vs**			
					**Type**	**Dose**	**Type**	**Dose**		**Measure**	**>Ex**	**>CT**	**>CON**
Legault et al., 2011	Community dwelling, risk of CI 70-85 years	80	Factorial ExCT, Ex, CT and Con	16	Memory training (before Ex)	50 min 1–2/week	Aerobic and flexibility	60 min 2/week (150 min total)	Health education	EF Mem	N N	N N	N N
Fabre et al., 2002	Community dwelling, 60–76 years	32	Factorial ExCT, Ex, CT and Con	8	Memory training (after Ex)	90 min 1/week	Aerobic	60 min 2/week	Health education	Mem	Y	Y	Y
van het Reve and de Bruin, 2014	Community/residentially dwelling ≥ 65 years	182	RCT ExCT and Ex	12	Attention and alertness training (unclear)	10 min 3/week	PRT and balance	30 min 3/week	–	EF RT/PS	Y N	– –	– –
Oswald et al., 2006	Community dwelling 75-93 years with 5-year follow-up	375	Quasi RCT Factorial ExCT, Ex, CT, PET, ExPET and Con	30	Processing speed, memory and attention training (before and after Ex)	90 min 1/week	Coordination flexibility and balance	45 min 1/week	No treatment	RT/PS Att Mem EF	NA NA NA NA	NA NA NA NA	Y Y Y Y
Barnes et al., 2013	Community dwelling, subjective CI ≥ 65 years	126	2 × 2 Factorial ExCT, CTCon, ExCon, Con	12	Visual and auditory processing training (unclear)	60 min 3/week	Aerobic and PRT	60 min 3/week	Educational DVDS, stretching and toning	EF VS Mem Global	N N N N	N N N N	N N N N
Shatil, 2013	Independent living- retirement village 65-93 years	180	Factorial ExCT, Ex, CT, and Con	16	Multidomain cognitive training (unclear)	40 min 3/week	Aerobic and PRT	45 min 3/week	Book club	Att Mem EF PS	N Y N Y	N N^#^ N N^#^	N Y N Y
Rahe et al., 2015	Community dwelling 50-85 years	68	Factorial ExCT^*^ CT	7	Memory, attention, EF training (after Ex)	90 min 2/week	Aerobic, PRT, coordination, flexibility	90 min 2/week	–	Mem Att EF Global VF	– – – – –	N N N N N	– – – – –
Desjardins-Crépeau et al., 2016	Community dwelling ≥60 years	76	2x2 Factorial ExCT, CTCon, ExCon, Con	12	DT cognitive training (after Ex)	60 min 1/week	Aerobic and PRT	60 min 2/week	Mental activity, stretching and toning	Mem EF Att	N N N	N N N	N Y^†^ N
Shah et al., 2014	Community dwelling 60-85 years	224	Non-RCT ExCT, CT, Ex, Con	16	Multidomain cognitive training (unclear)	60 min 5/week	Walking and PRT	60 min 3/week, 40 min 2/week	No treatment	Mem PS Att EF Global	N N N N N	N N N N N	Y N N N N
Linde and Alfermann, 2014	Community dwelling 60-75 years	70	Factorial ExCT, CT, Ex, Con	16	Multidomain cognitive training (before Ex)	30 min 1/week	Aerobic and PRT	60–90 min 2/week	No treatment	Att EF PS Mem	N N N N	N N N N	N N Y N

*Att, Attention; Con, Control group; CI, Cognitive impairment; CRT, Choice Reaction Time; CT, Cognitive training group; DT, Dual-task training; EF, Executive Function; Ex, Exercise group; ExCT, Exercise and Cognitive training combined group; Mem, Memory; Global, Global Cognitive Function; N, no; NA, not assessed; PET, Psychoeducational training; PRT, Progressive Resistance Training; PS, Processing speed; RCT, Randomized Controlled Trial; RT, Reaction time; VF, Verbal fluency; VR, Virtual Reality, VS, Visuospatial ability; Y, yes*.

† Only in DT training groups;

# *CT also greater than Con*.

There are several factors that could explain the mixed effects of sequential exercise-cognitive training on cognitive function, including variations in the dosage of physical and cognitive training provided, differences in exercise intensity and intervention lengths and the outcome measures and populations studied, the specificity of cognitive training (e.g., multi-domain vs. specific memory training), and/or whether the cognitive training was conducted prior to or after exercise training. For instance, a 16-week RCT in older adults observed no cognitive benefits when exercise training was delivered after the cognitive training (Legault et al., 2011). In contrast, a 30-week trial in adults aged 75–93 years reported significant cognitive improvements when exercise training was performed before cognitive training (Oswald et al., 2006). Evidence from animal studies indicates exercise promotes neurogenesis in the brain (Van Praag et al., 2005), while cognitive training regulates synaptic formation (Trachtenberg et al., 2002) and enhances the survival of these exercise-induced neurons (Fabel et al., 2009). Therefore, exercise may facilitate synaptic plasticity and neurogenesis within a fertile neuro-environment, created through the release of growth factors such as brain derived neurotrophic factor (BDNF) (Cotman et al., 2007). The potential cognitive advantages available from sequential training may therefore require an additional exposure to cognitive or motor training elements following exercise training, in order to exploit this environment to a greater extent than either cognitive or exercise training alone (Kraft, 2012; Curlik and Shors, 2013).

## Effects of simultaneous exercise-cognitive training on cognitive function

To date, no studies have directly evaluated the efficacy of sequential vs. simultaneous exercise-cognitive training on cognitive function in older adults. However, there is some evidence that simultaneous exercise-cognitive training studies incorporating aerobic training combined with cognitive challenges (e.g., exergaming) and memory training, are associated with cognitive improvements in healthy older adults (Anderson-Hanley et al., 2012; Theill et al., 2013; Barcelos et al., 2015). In addition, studies that have incorporated PRT with balance training (Hiyamizu et al., 2012), stepping exercises (Kayama et al., 2014; Nishiguchi et al., 2015), and/or aerobic training (Leon et al., 2015; Yokoyama et al., 2015), performed with secondary cognitive or motor tasks, have also shown positive effects on cognition (Table 2). For example, improvements in executive function, global cognitive function and attention were observed in community dwelling older adults following 12-weeks of low-intensity aerobic and PRT performed simultaneously with arithmetic and word games, compared to an exercise-only (60-min three times a week) group (Yokoyama et al., 2015). However, a limitation of many of the current simultaneous training interventions is a lack of a factorial 2 × 2 design, which makes it difficult to determine whether simultaneous exercise-cognitive programs can provide added cognitive benefits to exercise or cognitive training alone. Nonetheless, 12-weeks of a multimodal exercise program incorporating aerobic training and PRT (60-min twice per week) performed in conjunction with motor tasks (e.g., walking, object manipulation) regulated by external cues, improved choice reaction time in community-dwelling older adults, compared to a group that performed exercise only, and a usual care group (Leon et al., 2015). While further long-term, well-designed factorial RCTs are needed, the available data indicates that multimodal exercise programs performed under dual-task conditions provide more consistent cognitive benefits in older adults.

**Table 2:** Randomized controlled trials examining the effects of simultaneous (dual-task) exercise-cognitive training on cognitive function in older adults.

**Author**	**Participants**	**N**	**Study design**	**Duration (weeks)**	**Simultaneous intervention**			**Control group**	**Cognitive outcomes**	
					**Cognitive**	**Exercise**	**Dose**		**Measure**	**ExCT > Con**
Theill et al., 2013	Community-dwelling 65-84 years	63	RCT ExCT vs. CT vs. Con	10	Memory training	Aerobic training	ExCT, 40 min, 2/week (Total 800 min) CT, 30 min, 2/week	No treatment	EF Att Mem PS	Y^*^ N Y N
Anderson-Hanley et al., 2012	Independent living- retirement village ≥55 years	102	RCT ExCT vs. Ex	12	VR enhanced	Stationary cycling	45 min, 3-5/week (Total 2700 min)	–	EF Att VS Mem	Y N N N
Barcelos et al., 2015	Independent living- retirement village Mean age: 82 years	64	RCT ExCT (high) vs. ExCT (low)	12	VR enhanced high or low cognitive demand)	Stationary cycling	45 min, 3-5/week (Total 2700 min)	–	EF	Y
Hars et al., 2014	Community dwelling at risk of falling ≥65 years	134	RCT ExCT vs. Con	26	Auditory cues	Walking, motor activity	60 min, 1/week (Total 1,560 min)	No treatment	EF VS Global	Y N N
Marmeleira et al., 2009	Community dwelling ≥60 years	32	RCT ExCT vs. Con	12	Auditory and visual cues	Walking, motor activity	60 min, 3/week (Total 2,160 min)	No treatment	EF RT CRT Att VS	N Y N N Y
Leon et al., 2015	Community dwelling ≥60 years	151	RCT ExCT vs. Ex vs Con	12	Auditory and visual cues, motor activity	Aerobic (walking) and PRT	60 min, 2/week (Total 1,440 min)	No treatment	RT CRT	Y Y^*^
Schoene et al., 2013	Independent living- retirement village ≥65 years	37	RCT ExCT vs. Con	8	Computerized and interactive	Step training	20 min, 2-3/week (Total 480 min)	No treatment	PS EF	Y N
Schoene et al., 2015	Independent living- retirement village ≥60 years	90	RCT ExCT vs. Con	16	Computerized and interactive	Step training	20 min, 3/week (Total 960 min)	No treatment	EF PS CRT RT Att VS	N Y Y Y Y Y
Kounti et al., 2011	Community dwelling, with MCI ≥65 years	88	RCT ExCT vs. Con	20	Auditory and visual cues	Walking, motor activity, balance	90 min, 1/week (Total 1,800 min)	No treatment	Global Att VS EF Mem	Y Y Y Y N
Yokoyama et al., 2015	Community dwelling ≥65 years	27	RCT ExCT vs. Ex	12	Auditory cues, arithmetic and word games	Aerobic and PRT	60 min, 3/week (Total 2,160 min)	–	Global VF Att EF PS	N Y Y Y N
Nishiguchi et al., 2015	Community dwelling ≥60 years	48	RCT ExCT vs. Con	12	Verbal and motor tasks	PRT, step training, walking	90 min, 1/week (Total 1,080 min)	No treatment	Global Mem EF	N Y Y
Eggenberger et al., 2015	Community dwelling ≥70 years with 1-year follow-up	89	RCT ExCT (VR) vs. ExCT vs. Ex	26	Verbal memory training or VR enhanced	Aerobic (treadmill or dancing) + PRT and balance	60 min, 2/week (Total 3,120 min)	–	EF Mem Att PS	N N N N
Hiyamizu et al., 2012	Community dwelling ≥70 years	43	RCT ExCT vs. Ex	12	Arithmetic, visual, verbal training	PRT and balance	60 min, 2/week (Total 1,440 min)	–	EF	Y
Gill et al., 2016	Community dwelling 55–90 years, subjective CI	44	RCT ExCT vs. Ex	26	Verbal and arithmetic tasks	Aerobic, PRT, balance, flexibility, step training	90 min, 3/week (Total 1,440 min)	–	Global EF PS Mem VF	Y N N Y Y
Suzuki et al., 2012	Community dwelling, with MCI ≥65 years	50	RCT ExCT vs. Con	52	Verbal and memory tasks	Aerobic, PRT, balance, walking	90 min, 2/week (Total 80 sessions 7,200 min) Con: 3 times	Education	Global Mem PS VF EF	Y Y N Y N
Coelho et al., 2013a	Community-dwelling with AD (Mean age of >77.1 years)	27	RCT ExCT vs. Con	16	Auditory cues, motor, attention and EF training	Aerobic, PRT, balance, flexibility, agility training	60 min, 3/week (Total 2,880 min)	No treatment	EF Att	Y N
Maillot et al., 2012	Community dwelling older adults (Mean age 73.5 years)	32	RCT ExCT vs. Con	12		Aerobic within Exergaming	60 min, 2/week (Total 1,440 min)	No treatment	EF VS PS	Y Y Y
Kayama et al., 2014	Community dwelling ≥65 years	48	Unclear ExCT vs. Ex	12	Suduko within VR enhanced Tai-Chi	Aerobic, PRT, balance, flexibility, step training	80 min, 1/week (Total 960 min)	–	EF VF	Y N
Padala et al., 2012	Assisted living with mild AD ≥60 years	22	RCT ExCT vs. Ex	8		PRT, balance and yoga within Exergaming	30 min, 5/week (Total 1,200 min)	Walking group	Global	N

*AD, Alzheimer's disease; Att, Attention; Con, Control group; CI, Cognitive impairment; CRT, Choice Reaction Time; CT, Cognitive training group; DT, Dual-task training; EF, Executive Function; Ex, Exercise group; ExCT, Exercise and Cognitive training combined group; Global, Global Cognitive Function; MCI, mild cognitive impairment; Mem, Memory; N, no; PET, Psychoeducational training; PRT, Progressive Resistance Training; PS, Processing speed; RCT, Randomized Controlled Trial; RT, Reaction time; VF, Verbal fluency; VR, Virtual Reality, VS, Visuospatial ability; Y, yes*.

* *Compared to Ex and Con*.

To summarize the available data with regard to the effects of combined exercise-cognitive training on cognitive function in older adults, Zhu et al. (2016) conducted a meta-analysis of 20 intervention studies comprising 2,667 cognitively healthy older adults aged 65–82 years which included both sequential and simultaneous training studies. Overall, a small effect of these combined interventions (sequential and simultaneous together) on memory, executive function, attention, visuospatial ability and global cognition were observed when compared to a control group [effect size (ES) 0.29 (95% confidence interval (CI): 0.12–0.46), *P* < 0.001] or exercise training alone [ES: 0.22 (95% CI: 0.06–0.38), *P* < 0.01), but there were no differences between the combined intervention and cognitive training alone. Secondary analysis also revealed that the effect size for sequential interventions was less than those for simultaneous interventions [sequential ES 0.27 (95% CI: 0.08–0.46); simultaneous ES 0.43 (95% CI: 0.01–0.86)], but did not include exergaming or more recently published studies. Another review of 13 studies also reported that simultaneous dual-task training interventions including low-intensity activity (walking and balance training) performed with a secondary cognitive or motor tasks, can benefit both physical function (e.g., balance) and the cognitive abilities of older adults in dual-task situations (e.g., response time to an auditory cue while walking or balancing; Wollesen and Voelcker-Rehage, 2013). However, an important unanswered question is whether these benefits transfer to untrained cognitive abilities.

Exergaming is a relatively new and unique form of dual-task training that may provide an attractive alternative to traditional simultaneous training protocols for improving cognitive function. In contrast to more conventional simultaneous interventions that often use disparate training elements (e.g., walking while doing arithmetic), exergaming typically includes cognitive challenges embedded within realistic physical activities, whilst providing virtual feedback. One of the first RCTs of exergaming for older adults revealed a promising differential cognitive benefit of exergaming (pedaling a virtual reality enhanced stationary bike; *d* = 0.50 over and above traditional stationary cycling alone; Anderson-Hanley et al., 2012). A subsequent 2016 systematic review that focused on exergaming studies geared toward falls prevention included five RCTs and two uncontrolled studies, and advocated a role for exergaming in improving cognitive function, although did not find any additional cognitive benefits compared to exercise alone (Ogawa et al., 2016). However, this review only included two studies that had an appropriate control comparison group that received exercise alone, with one of these producing cognitive benefits (Kayama et al., 2014). Nevertheless, large effect sizes (η^2^ = 0.80) on cognitive function have been observed following a 12-week exergaming (Nintendo Wii) trial in one study involving 32 independent living older adults aged 65–78 years (Maillot et al., 2012). Computerized step-pad training, whereby participants take steps in different directions on a step pad following instructions displayed on a computer screen, has also been shown to provide some cognitive benefits in older adults (Schoene et al., 2013, 2015). For instance, an 8-week study in 37 older adults residing in independent retirement villages found that home-based step pad training 2–3 times per week for 15–20 min improved processing speed compared to normal aging (Schoene et al., 2013). Similarly, a 16-week study with more challenging stepping games improved central processing, visuospatial abilities, and efficiency of executive networks in 90 healthy older adults (Schoene et al., 2015). Although the number of studies is currently limited, the above findings provide some promising evidence that exergaming may improve cognitive function in older adults.

Cognitive improvement through simultaneous dual-task training may be shaped by the secondary attention-demanding task performed with exercise. Secondary tasks requiring a functional response to verbal and auditory cues (e.g., switching walking direction according to verbal instruction, stepping to music) or motor tasks requiring divided attention (e.g., tossing a ball while walking), have improved multiple cognitive abilities in older adults (Marmeleira et al., 2009; Kounti et al., 2011; Hars et al., 2014; Leon et al., 2015; Yokoyama et al., 2015). For instance, 6-months of stepping exercises (dancing) performed synchronously with verbal and motor tasks, in accordance to musical cues (60-min, once a week), led to significant improvements in executive function (and reduced the risk of falling) in community dwelling older adults at increased risk of falling, compared to usual care (Hars et al., 2014). However, simultaneous interventions incorporating memory training and arithmetic in secondary tasks have produced mixed findings (Hiyamizu et al., 2012; Theill et al., 2013; Eggenberger et al., 2015). For instance, a 26-week intervention combining PRT and balance training along with either treadmill walking performed simultaneously with verbal fluency training, or interactive stepping in adults over 70 years, had no effect on cognition compared to exercise alone (Eggenberger et al., 2015). Similarly, memory training with treadmill walking produced minimal improvements in various cognitive abilities (Theill et al., 2013). Collectively, these data suggest that secondary components which require the engagement of executive processes (e.g., inhibition, planning and execution of a rapid motor response), as required in responding to an external cue, may offer wider cognitive benefits when combined with exercise, compared to domain-specific cognitive tasks (e.g., memory training).

Currently there is a lack of available data to determine whether cognitively intact or impaired older adults may receive greater cognitive benefits from undertaking simultaneous exercise-cognitive training. A 2014 review considered simultaneous dual-task training to be effective for adults with cognitive impairment, however no dual-task studies involving cognitively healthy older adults were included for comparative effects (Law et al., 2014). Limited evidence from simultaneous studies with cognitively impaired older adults undertaking multimodal (Suzuki et al., 2012; Coelho et al., 2013a; Gill et al., 2016), and movement-based exercise training (Kounti et al., 2011) corroborates the cognitive benefits observed in healthy older adults. Whether simultaneous training is a viable strategy to preserve cognitive function in older adults with dementia or Alzheimer's disease (AD) is not known, but enhancements in abstract reasoning and attention were produced following a 16-week multimodal exercise program performed simultaneously with verbal and motor tasks (60-min, three times per week) in older adults with AD, compared to usual care (Coelho et al., 2013a). In contrast, no significant improvements in global cognitive function were observed in assisted living residents with mild AD who undertook 8-weeks of a multimodal exergaming program (PRT, balance and yoga 30 min per day, 5-times per week), compared to a walking group (Padala et al., 2012). Therefore, while simultaneous training may be beneficial in offsetting further cognitive decline in older adults with cognitive impairment, it is unclear whether this format may be effective for older adults with AD or dementia due to the limited data with discrepant intervention elements.

## Is there an optimal type, duration and dose of exercise-cognitive training?

An important question is whether there is an optimal training type and dose (frequency, intensity, duration) of simultaneous exercise and cognitive-motor training that promotes cognitive improvement in older adults. Based on the available data, interventions incorporating functional stepping exercises (Hars et al., 2014; Nishiguchi et al., 2015; Schoene et al., 2015; Gill et al., 2016), and moderate-intensity aerobic-based or multimodal exercise training (Anderson-Hanley et al., 2012; Suzuki et al., 2012; Theill et al., 2013; Coelho et al., 2013a; Barcelos et al., 2015; Gill et al., 2016) appear to be effective for improving specific cognitive domains in healthy and cognitively impaired older adults. In partial support of this notion, a systematic review of 20 RCTs found that five out of six studies that adopted a sequential approach and 12 out of 13 studies that implemented simultaneous training reported significant cognitive improvement, with the most successful interventions involving aerobic training and PRT, combined with cognitive training of attention, working memory or executive function (Lauenroth et al., 2016). However, this review did not separate the different types of combined training studies.

In terms of the optimal training dose, evidence from simultaneous training studies suggests that significant cognitive benefits for older adults typically appear after a minimum of 12-weeks or a total of 1,000 min (~16.5 h) of exposure, with a training frequency between 1 and 3 times per week (Table 2). However, significant improvements in cognitive function have been produced with less than 1,000 min of training (Theill et al., 2013; Schoene et al., 2015), but not with more than 3,000 min (50 h; Eggenberger et al., 2015). These inconsistencies may be due to heterogeneity in terms of the length of studies (7-weeks to 12-months), differences in training modalities and intensity, the unsystematic selection of cognitive assessments, and the characteristics of the control group. Despite these variations, 17 of the 19 simultaneous studies featured in Table 2 produced benefits in at least one cognitive domain, compared to six out of the ten sequential studies. Of further importance, simultaneous training minimizes the possibility for discrepant doses of training between intervention groups, as often seen in sequential training studies.

## Mechanism(s) underlying cognitive improvements following dual-task training

The neurophysiological mechanisms underlying the cognitive improvements observed through combined cognitive and exercise training are yet to be identified; however cognitive and exercise training may stimulate similar neurobiological processes which produce a synergistic response. Exercise and cognitive training both increase cerebral blood flow (Ide and Secher, 2000; Mozolic et al., 2010), and induce angiogenesis in the cortex and cerebellum (Black et al., 1990; Ding Y. H. et al., 2006). Similarly, both forms of training have demonstrated that executive control processes and underlying brain regions are plastic and adaptive in the aging brain (Erickson et al., 2007; Voelcker-Rehage and Niemann, 2013), in exerting training-induced plasticity (Colcombe et al., 2004; Erickson et al., 2007; Lustig et al., 2009) and increasing brain volume (Colcombe et al., 2006; Boyke et al., 2008), which correlate with better cognitive performance (Voss et al., 2010; Erickson et al., 2011). Emerging evidence suggests that these neural modifications may also occur following cognitively challenging exercise programs, as training-induced decreases in prefrontal brain activation have been detected following simultaneous dual-task exercise (Nishiguchi et al., 2015), and exergaming (Eggenberger et al., 2016), which have correlated with improvements in executive function, while increases in hippocampal volume (Rehfeld et al., 2017) and white matter integrity (Burzynska et al., 2017) have been shown following dancing training in older adults. This suggests that a combination of physical exercise, sensorimotor stimulation and cognitive engagement may facilitate neurophysiological changes that contribute to cognitive improvement. Exercise can also protect vasculature and neural tissue through counteracting age-related increases in circulating inflammatory biomarkers (Petersen and Pedersen, 2005; Cotman et al., 2007), which have been linked to cognitive decline and dementia (Yaffe et al., 2003; Engelhart et al., 2004). Emerging evidence has demonstrated that the exercise-induced release of growth factors such as BDNF and insulin-like growth factor (IGF-1) are important for neurogenesis, angiogenesis and synaptic plasticity (Ding Q. et al., 2006; Cotman et al., 2007). An increase in BDNF was observed following exergame cycling (Anderson-Hanley et al., 2012), which may be due to the addition of a cognitively challenging component (Pressler et al., 2015) as the effects of aerobic training on circulating BDNF in older adults are limited (Coelho et al., 2013b). Concentrations of amyloid-β (1–40) protein, a biomarker of AD, also increased within a group that performed multimodal training simultaneously with various secondary tasks (Yokoyama et al., 2015), and in a group that performed exercise alone. Further, a sequential exercise-cognitive program in community-dwelling adults (50–85 years) did not change concentrations of BDNF, IGF-1 or vascular endothelial growth factor (VEGF) (Rahe et al., 2015); however this may have resulted from an inadequate amount of time allocated for physical training in each session to elicit benefits. Future studies may determine whether sequential and simultaneous exercise-cognition training may activate different neurobiological pathways.

## Conclusion

Combined exercise and cognitive training interventions can improve cognitive function in healthy and cognitively impaired older adults, with evidence suggesting that simultaneous training may be more effective than sequential training and exercise alone. Moreover, 17 of the 19 simultaneous studies featured in this review produced benefits in at least one cognitive domain, compared to six out of the ten sequential studies reviewed. While the optimal characteristics of a simultaneous training program (e.g., type and dose of exercise and secondary tasks) remain to be determined, studies incorporating moderate-intensity multimodal training in combination with secondary tasks involving a functional response to sensory cues have improved executive abilities and memory, which may prolong functional independence in older adults. However, further research is needed to determine if these improvements can be maintained, and even prevent or delay the onset of cognitive impairment and dementia. Finally, an understanding of the neurobiological mechanisms underpinning any cognitive improvements available from combined exercise-cognitive training in older adults also requires further investigation.

## Author contributions

JT and RMD wrote the manuscript and RLD, CM, and LM reviewed the draft versions. All authors have read and approved the final version.

### Conflict of interest statement

The authors declare that the research was conducted in the absence of any commercial or financial relationships that could be construed as a potential conflict of interest. The reviewer BY and handling Editor declared their shared affiliation.

## References

[B1] Anderson-Hanley C. Arciero P. J. Brickman A. M. Nimon J. P. Okuma N. Westen S. C.. (2012). Exergaming and older adult cognition: a cluster randomized clinical trial. Am. J. Prev. Med. 42, 109–119. 10.1016/j.amepre.2011.10.016 22261206

[B2] Angevaren M. Aufdemkampe G. Verhaar H. Aleman A. Vanhees L. (2008). Physical activity and enhanced fitness to improve cognitive function in older people without known cognitive impairment. Cochrane Database Syst. Rev. 3:CD005381 10.1002/14651858.CD005381.pub3 18646126

[B3] Anstey K. J. Wood J. Lord S. Walker J. G. (2005). Cognitive, sensory and physical factors enabling driving safety in older adults. Clin. Psychol. Rev. 25, 45–65. 10.1016/j.cpr.2004.07.008 15596080

[B4] Barcelos N. Shah N. Cohen K. Hogan M. J. Mulkerrin E. Arciero P. J.. (2015). Aerobic and Cognitive Exercise (ACE) pilot study for older adults: executive function improves with cognitive challenge while exergaming. J. Int. Neuropsychol. Soc. 21, 768–779. 10.1017/S1355617715001083 26581789

[B5] Barnes D. E. Santos-Modesitt W. Poelke G. Kramer A. F. Castro C. Middleton L. E.. (2013). The Mental Activity and eXercise (MAX) trial: a randomized controlled trial to enhance cognitive function in older adults. JAMA Intern. Med. 173, 797–804. 10.1001/jamainternmed.2013.189 23545598 PMC5921904

[B6] Bharucha A. J. Pandav R. Shen C. Dodge H. H. Ganguli M. (2004). Predictors of nursing facility admission: a 12-year epidemiological study in the united states. J. Am. Geriatr. Soc. 52, 434–439. 10.1111/j.1532-5415.2004.52118.x 14962161

[B7] Black J. E. Isaacs K. R. Anderson B. J. Alcantara A. A. Greenough W. T. (1990). Learning causes synaptogenesis, whereas motor activity causes angiogenesis, in cerebellar cortex of adult rats. Proc. Natl. Acad. Sci. U.S.A. 87, 5568–5572. 10.1073/pnas.87.14.5568 1695380 PMC54366

[B8] Boyke J. Driemeyer J. Gaser C. Büchel C. May A. (2008). Training-induced brain structure changes in the elderly. J. Neurosci. 28, 7031–7035. 10.1523/JNEUROSCI.0742-08.2008 18614670 PMC6670504

[B9] Burzynska A. Z. Jiao Y. Knecht A. M. Fanning J. Awick E. A. Chen T.. (2017). White matter integrity declined over 6-months, but dance intervention improved integrity of the fornix of older adults. Front. Aging Neurosci. 9:59. 10.3389/fnagi.2017.00059 28360853 PMC5352690

[B10] Coelho F. G. Andrade L. P. Pedroso R. V. Santos-Galduroz R. F. Gobbi S. Costa J. L.. (2013a). Multimodal exercise intervention improves frontal cognitive functions and gait in Alzheimer's disease: a controlled trial. Geriatr. Gerontol. Int. 13, 198–203. 10.1111/j.1447-0594.2012.00887.x 22686565

[B11] Coelho F. G. Gobbi S. Andreatto C. A. Corazza D. I. Pedroso R. V. Santos-Galduroz R. F. (2013b). Physical exercise modulates peripheral levels of brain-derived neurotrophic factor (BDNF): a systematic review of experimental studies in the elderly. Arch. Gerontol. Geriatr. 56, 10–15. 10.1016/j.archger.2012.06.003 22749404

[B12] Colcombe S. J. Erickson K. I. Scalf P. E. Kim J. S. Prakash R. McAuley E.. (2006). Aerobic exercise training increases brain volume in aging humans. J. Gerontol. A Biol. Sci. Med. Sci. 61, 1166–1170. 10.1093/gerona/61.11.1166 17167157

[B13] Colcombe S. J. Kramer A. F. Erickson K. I. Scalf P. McAuley E. Cohen N. J.. (2004). Cardiovascular fitness, cortical plasticity, and aging. Proc. Natl. Acad. Sci. U.S.A. 101, 3316–3321. 10.1073/pnas.0400266101 14978288 PMC373255

[B14] Colcombe S. Kramer A. F. (2003). Fitness effects on the cognitive function of older adults: a meta-analytic study. Psychol. Sci. 14, 125–130. 10.1111/1467-9280.t01-1-01430 12661673

[B15] Cotman C. W. Berchtold N. C. Christie L.-A. (2007). Exercise builds brain health: key roles of growth factor cascades and inflammation. Trends Neurosci. 30, 464–472. 10.1016/j.tins.2007.06.011 17765329

[B16] Curlik D. Shors T. (2013). Training your brain: do mental and physical (MAP) training enhance cognition through the process of neurogenesis in the hippocampus? Neuropharmacology 64, 506–514. 10.1016/j.neuropharm.2012.07.027 22898496 PMC3445739

[B17] Desjardins-Crépeau L. Berryman N. Fraser S. A. Vu T. T. M. Kergoat M. J. Li K. Z.. (2016). Effects of combined physical and cognitive training on fitness and neuropsychological outcomes in healthy older adults. Clin. Interv. Aging 11:1287. 10.2147/CIA.S115711 27698558 PMC5034910

[B18] Ding Q. Vaynman S. Akhavan M. Ying Z. Gomez-Pinilla F. (2006). Insulin-like growth factor I interfaces with brain-derived neurotrophic factor-mediated synaptic plasticity to modulate aspects of exercise-induced cognitive function. Neuroscience 140, 823–833. 10.1016/j.neuroscience.2006.02.084 16650607

[B19] Ding Y. H. Li J. Zhou Y. Rafols J. A. Clark J. C. Ding Y. (2006). Cerebral angiogenesis and expression of angiogenic factors in aging rats after exercise. Curr. Neurovasc. Res. 3, 15–23. 10.2174/156720206775541787 16472122

[B20] Eggenberger P. Schumacher V. Angst M. Theill N. de Bruin E. D. (2015). Does multicomponent physical exercise with simultaneous cognitive training boost cognitive performance in older adults? a 6-month randomized controlled trial with a 1-year follow-up. Clin. Interv. Aging 10, 1335–1349. 10.2147/CIA.S87732 26316729 PMC4544626

[B21] Eggenberger P. Wolf M. Schumann M. de Bruin E. D. (2016). Exergame and balance training modulate prefrontal brain activity during walking and enhance executive function in older adults. Front. Aging Neurosci. 8:66. 10.3389/fnagi.2016.00066 27148041 PMC4828439

[B22] Engelhart M. J. Geerlings M. I. Meijer J. Kiliaan A. Ruitenberg A. van Swieten J. C.. (2004). Inflammatory proteins in plasma and the risk of dementia: the Rotterdam study. Arch. Neurol. 61, 668–672. 10.1001/archneur.61.5.668 15148142

[B23] Erickson K. I. Colcombe S. J. Wadhwa R. Bherer L. Peterson M. S. Scalf P. E.. (2007). Training-induced functional activation changes in dual-task processing: an FMRI study. Cereb. Cortex 17, 192–204. 10.1093/cercor/bhj137 16467562

[B24] Erickson K. I. Voss M. W. Prakash R. S. Basak C. Szabo A. Chaddock L.. (2011). Exercise training increases size of hippocampus and improves memory. Proc. Natl. Acad. Sci. U.S.A. 108, 3017–3022. 10.1073/pnas.1015950108 21282661 PMC3041121

[B25] Fabel K. Wolf S. Ehninger D. Babu H. Galicia P. Kempermann G. (2009). Additive effects of physical exercise and environmental enrichment on adult hippocampal neurogenesis in mice. Front. Neurosci. 3:9. 10.3389/neuro.22.002.2009 20582277 PMC2858601

[B26] Fabre C. Chamari K. Mucci P. Masse-Biron J. Prefaut C. (2002). Improvement of cognitive function by mental and/or individualized aerobic training in healthy elderly subjects. Int. J. Sports Med. 23, 415–421. 10.1055/s-2002-33735 12215960

[B27] Gill D. P. Gregory M. A. Zou G. Liu-Ambrose T. Shigematsu R. Hachinski V.. (2016). The healthy mind, healthy mobility trial: a novel exercise program for older adults. Med. Sci. Sports Exerc. 48, 297–306. 10.1249/MSS.0000000000000758 26285025

[B28] Hars M. Herrmann F. R. Gold G. Rizzoli R. Trombetti A. (2014). Effect of music-based multitask training on cognition and mood in older adults. Age Ageing 43, 196–200. 10.1093/ageing/aft163 24212920

[B29] Heyn P. Abreu B. C. Ottenbacher K. J. (2004). The effects of exercise training on elderly persons with cognitive impairment and dementia: a meta-analysis. Arch. Phys. Med. Rehabil. 85, 1694–1704. 10.1016/j.apmr.2004.03.019 15468033

[B30] Hiyamizu M. Morioka S. Shomoto K. Shimada T. (2012). Effects of dual task balance training on dual task performance in elderly people: a randomized controlled trial. Clin. Rehabil. 26, 58–67. 10.1177/0269215510394222 21421689

[B31] Ide K. Secher N. H. (2000). Cerebral blood flow and metabolism during exercise. Prog. Neurobiol. 61, 397–414. 10.1016/S0301-0082(99)00057-X 10727781

[B32] Kayama H. Okamoto K. Nishiguchi S. Yamada M. Kuroda T. Aoyama T. (2014). Effect of a Kinect-based exercise game on improving executive cognitive performance in community-dwelling elderly: case control study. J. Med. Internet Res. 16:e61. 10.2196/jmir.3108 24565806 PMC3961700

[B33] Kounti F. Bakoglidou E. Agogiatou C. Lombardo N. B. E. Serper L. L. Tsolaki M. (2011). RHEA,^*^ a nonpharmacological cognitive training intervention in patients with mild cognitive impairment. Top Geriatr. Rehabil. 27, 289–300. 10.1097/TGR.0b013e31821e59a9

[B34] Kraft E. (2012). Cognitive function, physical activity, and aging: possible biological links and implications for multimodal interventions. Aging Neuropsychol. Cogn. 19, 248–263. 10.1080/13825585.2011.645010 22313174

[B35] Lauenroth A. Ioannidis A. E. Teichmann B. (2016). Influence of combined physical and cognitive training on cognition: a systematic review. BMC Geriatrics 16:1. 10.1186/s12877-016-0315-1 27431673 PMC4950255

[B36] Law L. L. Barnett F. Yau M. K. Gray M. A. (2014). Effects of combined cognitive and exercise interventions on cognition in older adults with and without cognitive impairment: a systematic review. Ageing Res. Rev. 15, 61–75. 10.1016/j.arr.2014.02.008 24632497

[B37] Legault C. Jennings J. M. Katula J. A. Dagenbach D. Gaussoin S. A. Sink K. M.. (2011). Designing clinical trials for assessing the effects of cognitive training and physical activity interventions on cognitive outcomes: the seniors health and activity research program pilot (SHARP-P) study, a randomized controlled trial. BMC Geriatrics 11:27. 10.1186/1471-2318-11-27 21615936 PMC3126708

[B38] Leon J. Urena A. Bolanos M. J. Bilbao A. Ona A. (2015). A combination of physical and cognitive exercise improves reaction time in persons 61-84 years old. J. Aging Phys. Activ. 23, 72–77. 10.1123/JAPA.2012-0313 24413071

[B39] Linde K. Alfermann D. (2014). Single versus combined cognitive and physical activity effects on fluid cognitive abilities of healthy older adults: a 4-month randomized controlled trial with follow-up. J. Aging Phys. Activ. 22, 302–313. 10.1123/JAPA.2012-0149 23881448

[B40] Lustig C. Shah P. Seidler R. Reuter-Lorenz P. A. (2009). Aging, training, and the brain: a review and future directions. Neuropsychol. Rev. 19, 504–522. 10.1007/s11065-009-9119-9 19876740 PMC3005345

[B41] Maillot P. Perrot A. Hartley A. (2012). Effects of interactive physical-activity video-game training on physical and cognitive function in older adults. Psychol. Aging 27, 589–600. 10.1037/a0026268 22122605

[B42] Marmeleira J. F. Godinho M. B. Fernandes O. M. (2009). The effects of an exercise program on several abilities associated with driving performance in older adults. Accid. Anal. Prev. 41, 90–97. 10.1016/j.aap.2008.09.008 19114142

[B43] Martin M. Clare L. Altgassen A. M. Cameron M. H. Zehnder F. (2011). Cognition-based interventions for healthy older people and people with mild cognitive impairment. Cochrane Database Syst. Rev. 1:CD006220 10.1002/14651858.CD006220.pub2 21249675

[B44] Melzer I. Liebermann D. G. Krasovsky T. Oddsson L. I. (2010). Cognitive load affects lower limb force-time relations during voluntary rapid stepping in healthy old and young adults. J. Gerontol. A Biol. Sci. Med. Sci. 65, 400–406. 10.1093/gerona/glp185 19939911

[B45] Mozolic J. L. Hayaska S. Laurienti P. J. (2010). A cognitive training intervention increases resting cerebral blood flow in healthy older adults. Front. Hum. Neurosci. 4:10. 10.3389/neuro.09.016.2010 20300200 PMC2841485

[B46] Muir S. W. Gopaul K. Odasso M. M. M. (2012). The role of cognitive impairment in fall risk among older adults: a systematic review and meta-analysis. Age Ageing 41, 299–308. 10.1093/ageing/afs012 22374645

[B47] Nishiguchi S. Yamada M. Tanigawa T. Sekiyama K. Kawagoe T. Suzuki M.. (2015). A 12-week physical and cognitive exercise program can improve cognitive function and neural efficiency in community-dwelling older adults: a randomized controlled trial. J. Am. Geriatr. Soc. 63, 1355–1363. 10.1111/jgs.13481 26114906

[B48] Noack H. Lövdén M. Schmiedek F. (2014). On the validity and generality of transfer effects in cognitive training research. Psychol. Res. 78, 773–789. 10.1007/s00426-014-0564-6 24691586

[B49] Ogawa E. F. You T. Leveille S. G. (2016). Potential benefits of exergaming for cognition and dual-task function in older adults: a systematic review. J. Aging Phys. Activ. 24, 332–336. 10.1123/japa.2014-0267 26291754

[B50] Oswald W. D. Gunzelmann T. Rupprecht R. Hagen B. (2006). Differential effects of single versus combined cognitive and physical training with older adults: the SimA study in a 5-year perspective. Eur. J. Ageing 3, 179–192. 10.1007/s10433-006-0035-z 28794762 PMC5546372

[B51] Padala K. P. Padala P. R. Malloy T. R. Geske J. A. Dubbert P. M. Dennis R. A.. (2012). Wii-fit for improving gait and balance in an assisted living facility: a pilot study. J. Aging Res. 2012:97573. 10.1155/2012/597573 22745909 PMC3382377

[B52] Petersen A. M. Pedersen B. K. (2005). The anti-inflammatory effect of exercise. J. Appl. Physiol. 98, 1154–1162. 10.1152/japplphysiol.00164.2004 15772055

[B53] Pichierri G. Wolf P. Murer K. de Bruin E. D. (2011). Cognitive and cognitive-motor interventions affecting physical functioning: a systematic review. BMC Geriatrics 11:29. 10.1186/1471-2318-11-29 21651800 PMC3147016

[B54] Pressler S. J. Titler M. Koelling T. M. Riley P. L. Jung M. Hoyland-Domenico L.. (2015). Nurse-enhanced computerized cognitive training increases serum brain-derived neurotropic factor levels and improves working memory in heart failure. J. Card. Fail. 21, 630–641. 10.1016/j.cardfail.2015.05.004 25982826

[B55] Rahe J. Becker J. Fink G. R. Kessler J. Kukolja J. Rahn A.. (2015). Cognitive training with and without additional physical activity in healthy older adults: cognitive effects, neurobiological mechanisms, and prediction of training success. Front. Aging Neurosci. 7:187. 10.3389/fnagi.2015.00187 26528177 PMC4602086

[B56] Ratner E. Atkinson D. (2015). Why cognitive training and brain games will not prevent or forestall dementia. J. Am. Geriatr. Soc. 63, 2612–2614. 10.1111/jgs.1_13825 26660360

[B57] Rehfeld K. Müller P. Aye N. Schmicker M. Dordevic M. Kaufmann J.. (2017). Dancing or fitness sport? the effects of two training programs on hippocampal plasticity and balance abilities in healthy seniors. Front. Hum. Neurosci. 11:305. 10.3389/fnhum.2017.00305 28674488 PMC5475381

[B58] Schaefer S. Schumacher V. (2011). The interplay between cognitive and motor functioning in healthy older adults: findings from dual-task studies and suggestions for intervention. Gerontology 57, 239–246. 10.1159/000322197 20980735

[B59] Schoene D. Lord S. R. Delbaere K. Severino C. Davies T. A. Smith S. T. (2013). A randomized controlled pilot study of home-based step training in older people using videogame technology. PLoS ONE 8:e57734. 10.1371/journal.pone.0057734 23472104 PMC3589451

[B60] Schoene D. Valenzuela T. Toson B. Delbaere K. Severino C. Garcia J.. (2015). Interactive cognitive-motor step training improves cognitive risk factors of falling in older adults - a randomized controlled trial. PLoS ONE 10:e0145161. 10.1371/journal.pone.0145161 26673919 PMC4682965

[B61] Shah T. Verdile G. Sohrabi H. Campbell A. Putland E. Cheetham C.. (2014). A combination of physical activity and computerized brain training improves verbal memory and increases cerebral glucose metabolism in the elderly. Transl. Psychiatry 4:e487. 10.1038/tp.2014.122 25463973 PMC4270308

[B62] Shatil E. (2013). Does combined cognitive training and physical activity training enhance cognitive abilities more than either alone? a four-condition randomized controlled trial among healthy older adults. Front. Aging Neurosci. 5:8. 10.3389/fnagi.2013.00008 23531885 PMC3607803

[B63] Simons D. J. Boot W. R. Charness N. Gathercole S. E. Chabris C. F. Hambrick D. Z.. (2016). Do “brain-training” programs work? Psychol. Sci. Public Interest 17, 103–186. 10.1177/1529100616661983 27697851

[B64] Suzuki T. Shimada H. Makizako H. Doi T. Yoshida D. Tsutsumimoto K.. (2012). Effects of multicomponent exercise on cognitive function in older adults with amnestic mild cognitive impairment: a randomized controlled trial. BMC Neurol. 12:128. 10.1186/1471-2377-12-128 23113898 PMC3534485

[B65] Theill N. Schumacher V. Adelsberger R. Martin M. Jäncke L. (2013). Effects of simultaneously performed cognitive and physical training in older adults. BMC Neurosci. 14:103. 10.1186/1471-2202-14-103 24053148 PMC3856453

[B66] Tinetti M. E. Williams C. S. (1997). Falls, injuries due to falls, and the risk of admission to a nursing home. N. Engl. J. Med. 337, 1279–1284. 10.1056/NEJM199710303371806 9345078

[B67] Tinetti M. E. Doucette J. Claus E. Marottoli R. (1995). Risk factors for serious injury during falls by older persons in the community. J. Am. Geriatr. Soc. 43, 1214–1221. 10.1111/j.1532-5415.1995.tb07396.x 7594154

[B68] Trachtenberg J. T. Chen B. E. Knott G. W. Feng G. Sanes J. R. Welker E.. (2002). Long-term *in vivo* imaging of experience-dependent synaptic plasticity in adult cortex. Nature 420, 788–794. 10.1038/nature01273 12490942

[B69] Valenzuela M. Sachdev P. (2009). Can cognitive exercise prevent the onset of dementia? systematic review of randomized clinical trials with longitudinal follow-up. Am. J. Geriatr. Psychiatry 17, 179–187. 10.1097/JGP.0b013e3181953b57 19225276

[B70] van het Reve E. de Bruin E. D. (2014). Strength-balance supplemented with computerized cognitive training to improve dual task gait and divided attention in older adults: a multicenter randomized-controlled trial. BMC Geriatrics 14:134. 10.1186/1471-2318-14-134 25511081 PMC4293005

[B71] Van Praag H. Shubert T. Zhao C. Gage F. H. (2005). Exercise enhances learning and hippocampal neurogenesis in aged mice. J. Neurosci. 25, 8680–8685. 10.1523/JNEUROSCI.1731-05.2005 16177036 PMC1360197

[B72] Voelcker-Rehage C. Niemann C. (2013). Structural and functional brain changes related to different types of physical activity across the life span. Neurosci. Biobehav. Rev. 37, 2268–2295. 10.1016/j.neubiorev.2013.01.028 23399048

[B73] Voss M. W. Prakash R. S. Erickson K. I. Basak C. Chaddock L. Kim J. S.. (2010). Plasticity of brain networks in a randomized intervention trial of exercise training in older adults. Front. Aging Neurosci. 2:32. 10.3389/fnagi.2010.00032 20890449 PMC2947936

[B74] Wollesen B. Voelcker-Rehage C. (2013). Training effects on motor–cognitive dual-task performance in older adults. Eur. Rev. Aging Phys. A 11, 5–24. 10.1007/s11556-013-0122-z

[B75] Yaffe K. Lindquist K. Penninx B. Simonsick E. Pahor M. Kritchevsky S.. (2003). Inflammatory markers and cognition in well-functioning African-American and white elders. Neurology 61, 76–80. 10.1212/01.WNL.0000073620.42047.D7 12847160

[B76] Yokoyama H. Okazaki K. Imai D. Yamashina Y. Takeda R. Naghavi N.. (2015). The effect of cognitive-motor dual-task training on cognitive function and plasma amyloid beta peptide 42/40 ratio in healthy elderly persons: a randomized controlled trial. BMC Geriatrics 15:4. 10.1186/s12877-015-0058-4 26018225 PMC4446802

[B77] Zhu X. Yin S. Lang M. He R. Li J. (2016). The more the better? a meta-analysis on effects of combined cognitive and physical intervention on cognition in healthy older adults. Ageing Res. Rev. 31, 67–79. 10.1016/j.arr.2016.07.003 27423932

